# Meeting Proceedings

**Published:** 1854-03

**Authors:** 


*Meeting Proceedings.*

At a meeting of the Medical Class, Rush Medical College Session, 1853–4, called for the purpose of expressing the sentiments of the Class towards the Faculty, S. P. Yeomans, of Iowa, was called to the Chair, and J. F. Hamilton was elected Secretary.

On motion, the following Committee was appointed to draw up and report Resolutions. Drs. E. P. Wood, Root and Fish, who, after a short consultation, reported the following resolutions, which were unanimously adopted.

*Resolved*, That in taking leave of Rush Medical College, we unite with pleasure in expressing our entire approbation of the course of Medical Instruction which has been given during the past session.

*Resolved*, That while we feel grateful to all the members of the Faculty, for their unwearied efforts in our behalf, we would especially acknowledge our obligations to Prof. Herrick, who has, in an able and satisfactory manner, filled the chair of Surgery, to which he was so unexpectedly called, by the absence of his distinguished colleague, Dr. Brainard. Also to Drs. Freer and Johnson, who have filled the chairs of Anatomy and Physiology, for the untiring industry and ability which they have exhibited, and the skill which they have manifested, in rendering the instructions upon their respective branches to the fullest possible extent, complete and satisfactory.

*Resolved*, That a copy of the proceedings and resolutions be furnished to the city papers and the *N. W. Meeical and Surgical Journal*, for publication.

S. P. YEOMANS, President.

J. F. HAMILTON, Secretary.

Chicago, Feb. 21, 1854.

